# Selections

**Published:** 1816-03

**Authors:** 


### PART III.

### SELECTIONS.

### FRENCH DICTIONARY OF MEDICAL SCIENCES.*

*Article, docimasie pulmonaire. Pulmonum docimasia. Trial of the lungs*; from the Greek verb δοκιμαζω, I try—I essay.—Germ. *Lungen-probe.*—It. *Prova de' polmoni.*

To determine whether an infant has lived after birth, is the object of the various processes to which the respiratory organs of the new-born child have been at various times subjected. With respect to infanticide and the right of succession, it is a problem frequently discussed in the public tribunals; and constitutes, in its

* Our opinion having been more than once solicited on questions of child-murder, we deemed that an early notice of this article would be acceptable to our friends and Correspondents; and constitute a more satisfactory answer than we are able to deliver from personal observation and experience. The importance of the article, and the defect of any such minute and comprehensive view of the subject, in the English language, will best apologize for the length of the extract. Forensic medicine is a branch of science inexplicably slighted in Britain. We shall zealously exert ourselves to arouse the interest and attention of professional men towards it; and embrace every opportunity of illustrating the application of its principles to individual cases. We solicit from our friends, correct reports of trials for child-murder, poisoning, and contributions on any subject of medico-legal inquiry, that may come within their sphere of observation. —Edit.

relations to the former, one of the most delicate and important questions that can occupy the attention of the medico-legal inquirer. In proportion as philosophic scepticism has been introduced into our science, controversies have arisen respecting the validity of pulmonary trial: the confidence of the ancients in it been impugned; and its applicability to medicine, by some, utterly rejected. But the sober medium should be observed. The processes, which we have now to consider, although not infallible, are, under many circumstances, when properly performed and appreciated, capable of elucidating the obscurities of medico-legal research.

*Physiological theory, upon which pulmonary trial is founded.* The foetus cannot respire: little blood circulates in its pulmonary system; but, the connection between the mother and child destroyed, respiration becomes essential to the existence of the latter; hence, life and respiration should be regarded as inseparable points by the juridical physician, in all cases wherein, as upon questions of infanticide, the law requires proof of the existence not only of organic, but of animal life. Thus, every phenomenon, observed on the infant corpse, which indicates that respiration has taken place, displays equally the traces of life after birth. On this principle, pulmonary trial is instituted. Trial of *respiration* would be, perhaps, a more correct term; since our experiments and researches are not confined to the lungs, but extended to all the other organs which are interested in that function.

The changes, operated by respiration in the system of all mammiferous animals, are indeed not limited to the increase of specific lightness of the lungs, from introduction of air into their cells; or to the augmentation of their absolute gravity, from the perfect developement of their blood vessels. Other phænomena, equally conspicuous, succeed the respiratory act. These result, as well from the alterations of site, volume, and surface, in the apparatus of organs subservient to it, as from the new routes of circulation taken by the blood. Thus the lungs, before lying shrivelled at the bottom of the thorax, in colour obscurely red, in substance resembling the spleen, expand on the introduction of air, assume an emphysematous aspect and paler hue, fill the chest, and hide more or less the pericardium. Their blood becomes frothy; the thorax, once flat, vaulted; the tendinous centre of the diaphragm, depressed by inspiration, more deeply seated in the thoracic cavity; the foramen ovale, canalis arteriosus, and venosus, obliterated. The last three changes are not observed directly after birth. Time is essential to their production: their well-marked existence decidedly announces that respiration has taken place. The shrivelling of the navel-string, and establishment of the fæcal and urinary excretions, are appearances which, altho' less constant and direct, may, in combination with the preceding, tend to confirm the inference that a child has respired after birth.

*Description of the processes deduced from these data to ascertain whether respiration has taken place after birth.*—Whatever be the mode adopted to ascertain the condition of the lungs in these cases, we ought previously to examine the chest; whether it be raised, vaulted, or flattened and compressed. In opening its cavity, injury or derangement of the contained parts should be carefully avoided: their position, colour, and volume noted; the situation of the lungs, whether collapsed, compact, or dilated; and how far they cover the pericardium: whether, finally, the chest displays any morbid appearances. The value of these researches we shall elsewhere appreciate.

*Description of the hydrostatic trial of the lungs.* This process is of very old date. Galen slightly mentions it. Bartholin and Swammerdam revived it in 1664. It was first applied to forensic medicine by Schneger, in 1682. In making this experiment, we remove the lungs and heart from the chest, cutting away the trachea close to the former. The large vessels are previously secured. The blood on the exterior of the lungs sponged off, we place them gently in a sufficiently large vessel of water. This should be so deep as to afford a column of fluid proportioned both to the volume and weight of the heart and lungs. The water should be clean, of a middle temperature, and free from saline substances; which, by increasing its density, would counteract the gravitation of the lungs. Hence river-water is preferable. We have then to observe, whether the lungs and heart swim or sink; if they sink, whether suddenly or slowly. The experiment is to be repeated with the lungs separated from the heart. If one lung only swims, that lung should be distinguished. Then each lung should be tried separately, at first whole, afterwards cut into pieces; but the fragments of the right and left lung are not to be confounded. Each fragment should be pressed between the fingers, *under water*, to ascertain if bubbles of air escape; and whether, after this compression, it yet sink or swim. In cutting the substance of the lungs for this experiment, we should note if there be crepitation, consequent on the escape of air, which has been introduced into their cells by respiration or other means; whether their vessels contain much or little blood, and whether their internal structure be diseased. To these points we shall presently recur.

*Description of Plocquet's experiment by weight.*—Plocquet, in 1783, first made known this process, founded upon the following principle. On respiration, the pulmonary vessels are filled with blood: hence, in the infant which has respired, the relative weight, previously existing between the lungs and whole body, must be necessarily changed. Plocquet ascertained that the weight of the body of a male fœtus, *which had never respired*, was, including the lungs, 53040 grains, that of the lungs 792 grains; or, in other words, in the proportion of 67 to 1: in another *still-born* fœtus, as 70 to 1: while, in a third, prematurely born, *which had respired*, the proportion was 70 to 2. Thus the weight of the lungs is doubled by respiration: being in the fœtus, *previously to respiration*, as 1 to 70, in proportion to the body; and as 1 to 35, *after respiration has been effected.* The experiment of Plocquet then consists in weighing the whole body of the child before it is opened; and afterwards the lungs, separated from their connections and appendages; and lastly, comparing the whole weight of the body with that of the lungs.

*Description of Plocquet's experiment with the plumb line.*—To render more conclusive the preceding experiment, Plocquet recommends another; the results of both concurring, will decide more certainly the question of respiration after birth. This second has for its base the depression of the diaphragm towards the abdomen, consequent on inspiration. In the fœtus which has not respired, the inferior* surface of that muscle is much more convex than in the child which has respired. Hence, to ascertain precisely the degree of convexity, Plocquet prescribes the following rules: The abdomen previously opened, and all its appearances observed, we are cautiously to extract the viscera, in order to determine the situation of the diaphragm. 1st. By letting fall a plumb-line from the sternum, we must note the point of the thorax to which the tendinous centre of the diaphragm corresponds; and, when, by repeated experiment, this point has been fixed in the fœtus which has never respired; and the point to which the diaphragm is thrust downward, by respiration, we have obtained data capable of very useful application to our subject. 2dly. We should, moreover, ascertain whether the diaphragm may, or may not, be farther elevated towards the chest. In the former case, it is fair to presume, that the infant has respired; in the latter, that respiration has not taken place.

*Description of Daniel's experiment.*—This is founded on the increase of circumference acquired by the chest and lungs from respiration, and on the greater weight of the latter consequent on that act. He proposes, therefore, to measure the circumference of the chest, and compare it with the length of the dorsal portion of the spine; marking the distance from the sternum to the latter. The other part of the process, recommended by Daniel, is somewhat complex, and its description obscure. We shall transcribe it in the language of the French writer, Marc. "Every solid body, plunged into a liquid, displaces a quantity of such liquid proportioned to the space which it occupies. Hence the quantity displaced varies according to the volume of the solid body: an equal relation exists between them. Now, as the displaced liquid rises in the containing vessel, we conclude, that such elevation is exactly proportioned to the volume of the solid body immersed; thus, by placing a (graduated) scale in the vessel, the volume of the lungs may be numerically determined. The air introduced into the lungs by inspiration is never wholly expelled: consequently, on this increase of volume, they displace much more liquid; and the latter rises farther in the vessel when respiration has, than when it has not, taken place. If the relation between

---

* This surely should be read *superior*. We have before corrected a nearly similar error.—Edit.

lungs which have respired, and those which have not, be established on these principles, we can, by immersion of the lungs in a liquid, determine whether or not they contain air. Yet, as the presence of air in the lungs does not certainly decide the question of respiration, the experiment just described would be insufficient, if not supported by another. We know that every solid body, immersed in a liquid lighter than itself, loses in weight as much as the volume of liquid equal to that of the solid body would weigh; and that the weight of the liquid augments in like proportion. Consequently, bodies of the same weight, but different volume, plunged in the same liquid, must sustain an unequal loss of weight; and this loss will be greater in the more voluminous body, and the increase of weight of the liquid will, in like manner, be more considerable. Now, as the lungs of the fœtus, which has respired, are more voluminous than those of the fœtus which has not respired, the first will lose most weight in the liquid. The point then is, to establish on both descriptions of fœtus, on those also into whose lungs air has been artificially introduced, or extricated by putrefaction, the different relations of this loss to the specific gravity of the lungs. So we may decide, by comparison, whether respiration has existed or not."

" For example, let the diminution of the weight of the

| | |
|---|---|
| lungs, distended by respiration, be stated at - - - - - | 5 |
| ——, inflated after death - - - - - - - - - - - - - | 4 |
| —— of still-born children - - - - - - - - - - | 2 |
| ——, distended by putrefaction - - - - - - - - - - | $2\frac{1}{3}$ |

On the other hand, let the specific gravity of the

| | |
|---|---|
| Lungs of still-born children be - - - - - - - - - - | 6 |
| —— of children cut off by hæmorrhage - - - - - - | 5 |
| —— in a state of putrefaction - - - - - - - - - - | $5\frac{1}{2}$ |
| —— inflated after death - - - - - - - - - - - - | $5\frac{1}{2}$ |
| —— after respiration - - - - - - - - - - - - | 8 |
| —— of children asphyxiated - - - - - - - - - - | 9 |

We shall find that the volume of lungs which have neither been augmented by inflation, nor putrefaction, will be, to their specific gravity, in

| | | | |
|---|---|---|---|
| Children, still-born, as - - - - - - - - - - | 2 | to | 6 |
| ————, cut off by hæmorrhage - - - - - - | 2 | — | 5 |
| ————, the lungs in a state of putrefaction - - | $2\frac{1}{3}$ | — | $5\frac{1}{2}$ |
| ————, ———— inflated after death, 4 to 6 or | 4 | — | $5\frac{1}{2}$ |
| ————, ———— having respired - - - - - | 5 | — | 8 |
| ———— asphyxiated - - - - - - - - - - | 5 | — | 9" |

" To execute this experiment, we must proceed thus: The lungs and heart are to be removed from the chest; the great vessels tied to prevent the introduction of water. The absolute weight of these organs is then to be ascertained by placing them in very correct and delicate scales. Next, without withdrawing them from the balance, they must be plunged into a vessel of water sufficiently deep, that the loss of weight may be determined. Lastly, the heart should be separated from the lungs, and the latter again immersed, that the weight of the heart may be deducted. Yet, as the lightness of the lungs, when filled with air, would prevent their sinking, and thus mar the experiment, it will be necessary to affix to them a body, the weight of which is known, that it may again be deducted. Daniel recommends for this purpose a small basket, of silver wire, in which the lungs may be placed. A graduated glass tube may also be fixed along the internal surface of the vessel destined to contain the water, by which the degree of elevation of that fluid will be indicated. Thus both processes, of which the experiment is composed, may be executed at once."

*Appreciation of the different means tending to ascertain whether respiration has taken place after birth.—Appreciation of the inferences which may be drawn from the degree of arching of the chest.* Daniel attached great importance to this sign: he regarded its existence as a certain proof that respiration had taken place. Some have pretended that the faculty of determining, from external inspection alone, whether a child has respired, may be attained by long habit. The irregularities which the conformation of the chest displays in different subjects, and the difficulty of judging by approximation, conspire to render this procedure extremely fallacious. Isolately considered, this sign then is not entitled to confidence: taken, nevertheless, in combination with others, it may be advantageously admitted to decide the question of respiration.

*Appreciation of the inferences which may be drawn from the situation and volume of the lungs.* The opinion, generally received in forensic medicine, that the lungs of the infant which has respired, are so dilated as to cover the pericardium, is founded on the view of subjects in whom perfect respiration has been, for some time, established. But some anatomists, particularly Schmitt, have observed, that in children dying soon after birth, even with a free respiration, the pericardium was not wholly covered by the lungs. In most cases, it is covered by the right lung; in a very few others, almost wholly by both: yet this appearance will not exist unless respiration have gone on several days. It is then useful to state more precisely than writers have generally done, the phænomenon in question, and adopt the principles of those only who have represented it aright.* In fact, according to the experiments of

* Ejus enim qui non respiravit, pulmones collapsi ad dorsi vertebras conspiciuntur, pectoris cavum non omninò explent *nec pericardium adeo tegunt*: ejus verò qui respiravit, pulmones pectoris cavum implent, magisque pericardiam abscondunt. *Daniel. Commentatio, &c. page* 186.—Respiraverit ne infans natus pulmones docere possunt, si nimirum ii densi versus dorsum retracti—nec expansi sint, *ac pericardium a pulmone sinistro haud tectum sit, sed nudum conspicitur*......Tunc in fœtu nullam a partu

Schmitt, there exists, in this respect, only a relative difference between the fœtus which has respired, and that which has not. The right side of the pericardium is commonly more covered than the left, because the right lung is more voluminous than its fellow; and respiration sooner and more energetically established in it. Yet this rule is not without exceptions; which, though rare, suffice to invalidate it as a general principle, and render this phænomenon inconclusive with respect to the question of respiration after birth. The space occupied by the lungs in the chest is generally subordinate to their volume; and, although this last be doubtless relative to the degree of expansion of the pulmonary organ, there may exist variations which it is necessary to recollect. Thus, in four experiments of Schmitt, the lungs of the fœtus which had not respired, filled the whole thoracic cavity; while, in another, which had respired thirty-six hours, the lungs, though filled with air, were so small that they could not at first be discovered.

*Appreciation of the inferences which may be drawn from the colour of the lungs.* The colour of the lungs which have not respired, is commonly brown or violet; rose-like, when respiration has been effected; but this sign is not entitled to unlimited confidence. No organ is more capable of assuming various shades of colour; and the tint not only may differ, according to the different degrees of respiration, but may be modified by the influence of many causes internal and external. Thus, the hue of the pulmonary surface, especially when deep, changes soon after opening the chest, and becomes clearer from the mere action of atmospheric air. Again, the colour of the lungs in still-born children, sometimes resembles so little that commonly met with, as to deceive the most experienced observer. This peculiarity is more frequent in proportion as the fœtus is less advanced; and the greater relative developement of the lungs at an early period, adds to the delusion. Lastly, the lungs of children, destroyed by suffocation, or into whose thorax blood has been effused, sometimes exhibit a brown colour; and hence a presumption might arise that respiration had not taken place. Yet all these different exceptions are rare; and the colour of the lungs, although, when considered isolately, of little importance, becomes conclusive in combination with other proofs which establish, or controvert, the existence of respiration.

*Appreciation of the inferences which may be drawn from the state of the canalis arteriosus, and foramen ovale, the canalis venosus, and umbilical cord.* The alterations which these parts undergo in consequence of respiration, do not take place until that function has been, for some days, established; and, as infanticide is

---

respirationem adfuisse concludendum. Contrà ubi uterque pulmo expansus pectoris cavitates replevit, atque *a superiore sinistra parte,* pericardio suo modo tectum est....tunc fœtum a partu respirasse, adeòque vixisse, constat. *Sikora, Consp. Med. legalis.*

commonly perpetrated immediately after birth, we have rarely an opportunity of observing these alterations, which, when they exist, constitute the strongest proof that respiration has been accomplished.

*Appreciation of the inferences which may be drawn from the state of the intestines and bladder.* Respiration, by thrusting downward the diaphragm and abdominal viscera, induces evacuation of the intestines and bladder; and, from their emptiness or plenitude, it has been attempted to establish the existence, or absence, of the respiratory act after birth. But no sign can be more equivocal than this: for there are many causes which may determine evacuation of the meconium and urine before birth, or retard these excretions after respiration has been effected. Nevertheless, the condition of these organs should always be ascertained and reported, as an accessory proof, in combination with other signs.

*Appreciation of the old hydrostatic trial.* The *first objection* raised against this process is, that *a fœtus may respire before birth, and afterwards perish during expulsion.* If, by respiration before birth, be meant the justly disputed phænomenon, called *vagitus uterinus*, the crying of the fœtus while yet enclosed within its membranes, nothing, worthy of confidence has been advanced in proof of it. The analogy of the chick, which is heard to chirp before extrication from the shell, can here have no weight. The shell, extremely porous, impedes not, like the membranes of the human fœtus, the introduction of the external air. The chick, moreover, is an isolated being; and, having no communication with its mother, may experience a real necessity of respiring when ready to break the shell. But this can never happen to the fœtus of mammiferous animals.

The objection, however, assumes another aspect, if, by respiration after birth, we are to understand the performance of that act, when the head of the fœtus, after rupture of its membranes, has been impelled towards the external parts of the mother, and rests sufficiently long in this position for the mouth and nostrils to be exposed to the immediate contact of the air. Nothing has been neglected that might solve or elucidate a question, thus interesting to the doctrine of pulmonary trial. Osiander asserts, that the fœtus may respire and cry, when the *liquor amnii* has escaped, and its mouth is near the uterine orifice. Our author, Marc, completely disbelieved the fact, until Beclard,* upon opening carefully the womb of a pregnant animal, observed, through the membranes, very distinct respiratory movements, repeated at regular intervals, and generally slower than those of the extra-uterine life of the same animal. They are more frequently performed as the circulation between the mother and fœtus becomes, from progressive contraction of the womb, more imperfect. They closely resemble

* Chef des travaux anatomiques de la Faculté de Médecine de Paris.

the deep and distant efforts of respiration, which take place in the *asphyxia of new-born children* This remark confirms, in some degree, the assertion of Osiander. Another question, no less important than the former, and warmly controverted by juridical physicians, presents itself: Can the fœtus, whose head alone has passed the os externum, respire, while the remainder of its body is yet enclosed by the sexual parts of the mother? Some eminent men, among whom may be enumerated Camper, Roederer, Wrisberg, Meckel, Daniel, and Metzger, have resolved it in the negative: others, as Bohn, Haller, Morgagni, Teichmeyer, Plocquet, Roose, and Schmidtmuller, have taken the opposite side; admitting, under these circumstances, the possibility of imperfect respiration.

Our author enters somewhat diffusely into this question. He, at first, adopted the arguments and opinions of Camper; but subsequently appears to admit, with great candour, the affirmative evidence of Dr. Hunter, Baudelocque, Osiander, and Schmitt. Osiander records nine cases, in which respiration had taken place, either after the protrusion of the head only, or during the passage of the thorax: in three of these, the umbilical cord was twisted round the child's neck. The cases, observed by Schmitt, constitute a conclusive testimony on this subject: he was directed by impartiality and love of truth. These cases are eight in number. Of the three, selected by Marc, we transcribe the last, and perhaps the best. "On the 28th of December, 1804, I delivered a young lady of distinction of her first child The straitness and obstinate resistance of the genital parts considerably embarrassed and retarded the passage of the head of the fœtus. Scarcely had it protruded, when the infant began to respire and cry, although the umbilical cord made two turns around the neck. The shortness of the cord, the breadth of the shoulders, and cessation of the pains, prevented the expulsion of the trunk, which was disengaged but slowly; so that the chest, abdomen, and feet, were delivered at distinct intervals. The infant continued to respire all this time, and frequently uttered a kind of cry. Several persons witnessed this occurrence; among others, M. de Vering, physician-in-chief of the armies." Schmitt adds, that he not only heard, but had ocular demonstration of, the circumstances which he records.

Can the infant, all whose parts except the head are expelled, respire, when, by the efforts of the hand of the accoucheur to disengage it, air has been introduced into the vagina? Upon this point much difference of opinion has existed. Osiander affirms, that, after having turned a child, he supported its body with his right arm, till the head should be extricated; and that, on passing his hand beyond the mouth to the upper jaw, the infant moved its shoulders, and the thorax performed the act of respiration. The sixth and twenty-third cases of Schmitt apparently confirm the opinion of Osiander. In one, the fœtus was at its full time, but delivery was expedited on account of dangerous uterine hæ-

morrhage. The child, extracted by turning, displayed no sign of life. The state of the lungs denoted imperfect respiration: they occupied less the posterior part of the chest; their colour was less deep, their substance more spongy, than in children who have not respired. They swam in water with and without the heart, although incompletely: the inferior lobes particularly had a tendency to sink. The substance of the organ was crepitous, and fragments of it yielded, on pressure, a white froth inclining to red. The subject of the other case was a male fœtus of eight months, which perished during the operation of turning, because of the extreme difficulty encountered in disengaging the head. The lungs, connected with the heart, sank, but slowly; so that the latter touched the bottom of the vessel, while the lungs rose towards the surface. Separated from the heart, they sank; but directly rose again, and remained at the surface of the liquid. The same phænomenon took place with each lung separately, as well as with their lobes, except that the middle right lobe, and superior left lobe, sank slowly, and did not rise again. The consistence of the lungs was less compact than when respiration had not existed; their colour hepatic, but, in many places, especially near the borders, clear and verging towards pink. When cut in pieces, much white froth could be expressed: they contained a considerable quantity of blood.

"From what has been just said, it results, that, in the actual state of our knowledge, the possibility of the fœtus respiring before birth must be admitted; and that this truth, applied to experiments on respiration of every kind, constitutes a restriction by no means to be lost sight of, whenever, from the phænomena which such experiments exhibit, the question of respiration, and absolute life, after birth, is to be decided."

Four other *objections* to the hydrostatic trial of the lungs are stated, and discussed at considerable length, by the French writer. With an abridgment of these, and his final conclusions, we shall terminate this interesting article in our next Number. Edit.

### *Case of Sphacelation of the Ileum.**

"In the month of August, 1814, a woman, of Genevilliers, near Paris, aged fifty years, married a second husband. The family, on the man's side, was much dissatisfied with this union. Five days subsequently to the marriage, the woman, after having drunk a cup of coffee with her husband and one of his nieces, was attacked with violent colics and great efforts to vomit. Diluent drinks and injections were liberally administered: the symptoms,

* Read at La Société de Médecine-Pratique, by Léon-Gagné, M. D. of Neuilly. See Journal de Médecine, &c. par Leroux, for September, 1815. This case, so interesting in its relations to forensic medicine, we have thought worthy of a place in our Selection department. Edit.

nevertheless, were progressely aggravated, and the poor woman died after twenty-six hours. The husband was suspected of having poisoned her; and, incited by the public clamour, the magistrate requested me to inquire into the death of the woman.

"On arrival at the house, I desired to see the matter discharged by stool and vomiting: they could shew me nothing.

"*Dissection of the body.*—The face, neck, and breast, were of a livid brown colour; the abdomen much swollen *(ballonné)*, presented several livid spots.

"On opening the abdomen, a great quantity of reddish and inodorous fluid escaped. The omentum was brown and injected with blood. The stomach and intestinal tube were distended; and their capillary vessels gorged with black blood. The internal membrane of the stomach was injected with brown-coloured blood, and shewed no vestige of erosion. On tracing the intestinal tube, a portion of the ileum, in the right iliac cavity, was found filled with a hard substance, of the size of a large goose-egg; and the intestine black and sphacelated to the extent of about eight inches. Below this point a *strangulation* existed, and many adhesions to the cellular membrane of the iliac cavity.

"From the intelligence which I was able to procure, it seems that this woman complained frequently of colic of the right side, and even vomited fæcal matter. Hence a contraction in that part of the *ileum intestinum*, to which I allude, must have long existed: and the immediate cause of the death of the woman may be satisfactorily explained. From the period of her marriage, she had abandoned her usual regimen, which consisted of light food, and had neglected to preserve the freedom of the bowels by injections. The consequence of this imprudent conduct was accumulation and hardening of the contents of the intestines; inflammation, gangrene, and death."

